# Local Consolidative Surgery for Oligometastatic Non-Small Cell Lung Cancer With Bone Metastasis

**DOI:** 10.1016/j.atssr.2025.09.022

**Published:** 2025-10-23

**Authors:** Zamaan Hooda, Shanique Ries, Michael Eisenberg, Raphael Werner, Wayne Hofstetter, Reza Mehran, Ravi Rajaram, David Rice, Stephen Swisher, Ara Vaporciyan, Garrett Walsh, Isabelle Opitz, Kyle G. Mitchell, Mara B. Antonoff

**Affiliations:** 1 Department of Thoracic and Cardiovascular Surgery, University of Texas MD Anderson Cancer Center, Houston, Texas; 2 Department of Thoracic Surgery, University Hospital Zurich, Zurich, Switzerland

## Abstract

**Background:**

Local consolidative therapy (LCT) can improve overall survival (OS) and progression-free survival (PFS) in oligometastatic non-small cell lung cancer (NSCLC). Bone metastases (BM) are associated with poorer prognosis after LCT. However, the prognostic influence of BM on outcomes after pulmonary resection remains unknown, particularly in an era with advanced systemic therapies.

**Methods:**

We identified patients from 2 centers with oligometastatic (≤3 synchronous sites) NSCLC who underwent pulmonary resection from 1996 to 2023. Patients were stratified by BM presence. Survival outcomes were evaluated by Kaplan-Meier and multivariable Cox regression models.

**Results:**

There were 87 patients who met study criteria, including 10 (11.5%) patients with BM (+BM) and 77 (88.5%) without (−BM). Age and smoking patterns were not statistically different between groups, and median number of metastatic sites was 1.0 (interquartile range, 0) for all. Notably, median OS was similar between groups, 39.2 months for +BM (95% CI, 11.81-66.51) and 37.5 months for −BM (95% CI, 22.92-51.98; *P* = .45). Median PFS was likewise similar, 17.7 months for +BM (95% CI, 5.13-30.29) and 18.4 months for −BM (95% CI, 11.86-24.86; *P* = .28). Bone involvement did not independently predict PFS (hazard ratio, 1.45; 95% CI, 0.74-2.82; *P* = .28) or OS (hazard ratio, 1.29; 95% CI, 0.66-2.51; *P* = .45).

**Conclusions:**

In this contemporary study of patients with oligometastatic NSCLC undergoing pulmonary resection as part of comprehensive LCT, +BM patients were not observed to have poorer survival outcomes. Multidisciplinary teams should consider aggressive LCT approaches including pulmonary resection in this setting.

**In Short**

▪ This multicenter study of patients with oligometastatic non-small cell lung cancer found that bone metastatic disease did not have an impact on survival and oncologic outcomes after pulmonary resection as part of comprehensive local consolidative therapy.
▪ Bone metastases in the setting of oligometastatic non-small cell lung cancer should not dissuade clinicians from pursuing aggressive local consolidative therapy strategies with pulmonary resection in appropriately selected patients.

The efficacy in controlling distinct disease sites in oligometastatic (≤3 synchronous metastases) non-small cell lung cancer (NSCLC) with surgical or radiation therapy, termed local consolidative therapy (LCT), has shifted management paradigms.^1^, ^2^, ^3^ Systemic agents have improved overall survival (OS), increasing the importance of integrating LCT into the care of these patients.^1^

Previously, we showed that comprehensive LCT (cLCT) is associated with improved OS in oligometastatic NSCLC, although these benefits may be limited in patients with bone metastases (BM).^2^ However, these patients received heterogeneous cLCT modalities that included cytotoxic chemotherapy, and the prognostic influence of BM in those specifically undergoing pulmonary resection remains unclear. We aimed to ascertain the impact of BM on oncologic and survival outcomes in patients with oligometastatic NSCLC undergoing pulmonary resection in a contemporary era. We hypothesized that BM may be less relevant with advanced therapies.

## Material and Methods

We retrospectively reviewed data from 2 institutions. Eligible patients had confirmed oligometastatic NSCLC; were treated between January 1, 1996, and December 31, 2023; and underwent pulmonary resection as part of cLCT.^2^, ^3^, ^4^ TNM staging followed the eighth edition of the American Joint Commission on Cancer staging manual.^5^ Cohort identification was derived from natural language processing methods,^1^ and patients were stratified by BM.

Definitions reflected those used in prior research.^2^, ^3^, ^4^ Intrathoracic nodal disease, irrespective of the number of involved lymph nodes, was counted as 1 metastatic site, and multiple metastases within an organ were counted as distinct metastases.^1^ BM presence was categorized as any lesion within the bony skeleton. cLCT referred to combining LCT modalities directed at both the primary tumor and metastatic sites.^1^ OS measured time from pulmonary resection to death, and progression-free survival (PFS) measured time from resection to progression or death without progression. Patients alive or dead without progression at the study’s end were censored at last follow-up.

Categorical variables were analyzed by Pearson *χ*^2^ and Fisher exact tests; continuous variables were analyzed with Mann-Whitney *U* test. OS and PFS were analyzed by the Kaplan-Meier method and the log-rank test. Multivariable Cox regression analysis evaluated the unadjusted and adjusted influences of clinicopathologic variables on OS and PFS. Univariate variables included age, sex, smoking history, number and location of metastases, BM presence, histologic grading, resection margins, and lymphovascular invasion. Analyses were performed with IBM SPSS Statistics (version 24). Two-sided *P* values < .05 were considered statistically significant.

## Results

### Population of Patients

Eighty-seven patients met inclusion criteria: 61 (70.1%) from MD Anderson Cancer Center and 26 (29.9%) from the University of Zurich (Table 1). Of these patients, 10 (11.5%) had BM and 77 (88.5%) did not. Both groups had similar median age at surgery (BM, 58 [interquartile range, 11.34] years; non-BM, 58.3 [interquartile range, 14.17] years). The BM and non-BM groups had 3 (30%) and 41 (53.2%) female patients, respectively. Half (50%) of the BM group had clinical tumor status (cT) of cT2, and cT1 was the most common in non-BM patients (44.1% [n = 34/77]; Table 1). Most patients had clinical nodal status (cN) of cN0 (BM, 50% [n = 5/10]; non-BM, 58.4% [n = 45/77]).

**Table 1:** Demographic and Clinicopathologic Information

Variable	Bone Metastases (n = 10)	No Bone Metastases (n = 77)	*P*
Age at surgery, y, median (SD)	58.0 (±15.1)	58.3 (±10.1)	.608
Female sex	3 (30)	41 (53.2)	.167
Former/current smoker	7 (70)	64 (83.1)	.314
Race/ethnicity			.482
White	8 (80)	68 (88.3)	
Black	1 (10)	4 (5.2)	
Hispanic	1 (10)	3 (3.9)	
Asian	0	1 (1.3)	
Primary tumor location			
Right upper lobe	4 (40)	28 (36.4)	1
Right middle lobe	0	4 (5.2)	1
Right lower lobe	2 (20)	13 (16.9)	.681
Left upper lobe	3 (30)	20 (25.9)	.720
Left lower lobe	1 (10)	12 (15.6)	1
Unspecified	1 (10)	0	.115
No. of metastatic lesions			.004
1	7 (70)	76 (98.7)	
2	2 (20)	1 (1.3)	
3	1 (10)	0	
Metastatic sites			
Bone	10 (100)	0	0
Brain	2 (20)	51 (66.2)	.013
Adrenal	1 (10)	5 (6.5)	.530
Contralateral lung	0	13 (16.9)	.180
Pleura	1 (10)	6 (7.8)	.588
Other	0	5 (6.5)	0
cT			.056
1	2 (20)	34 (44.1)	
2	5 (50)	26 (33.8)	
3	3 (30)	6 (7.8)	
4	0	11 (14.3)	
cN			.703
0	5 (50)	45 (58.4)	
1	3 (30)	12 (15.6)	
2	2 (20)	17 (22.1)	
3	0	3 (3.9)	

Categorical variables are presented as number (percentage).

cN, clinical nodal status; cT, clinical tumor status.

Most patients received neoadjuvant treatment with chemotherapy, immunotherapy, targeted therapy, or a combination of these agents (BM patients, 80% [n = 8/10]; non-BM patients, 53.2% [n = 41/77]; Table 2). Fewer BM patients (40% [n = 4/10]) than non-BM patients (84.4% [n = 65/77]) underwent resection of metastatic lesions, whereas radiotherapy use as LCT was similar (BM patients, 80% [n = 8/10]; non-BM patients, 71.5% [n = 55/77]).

**Table 2:** Treatment, Operative, and Postoperative Details

Variable	Bone Metastases (n = 10)	No Bone Metastases (n = 77)	*P*
Neoadjuvant therapy			.175
None	2 (20)	36 (46.8)	
Chemotherapy	3 (30)	31 (40.2)	
Immunotherapy	0 (0)	0 (0)	
Targeted therapy	4 (40)	5 (6.5)	
Chemotherapy + targeted therapy	0 (0)	4 (5.2)	
Chemotherapy + immunotherapy	1 (10)	1 (1.3)	
Adjuvant therapy			.503
None	3 (30)	35 (45.4)	
Chemotherapy	2 (20)	25 (32.5)	
Immunotherapy	1 (10)	0 (0)	
Targeted therapy	4 (40)	14 (18.2)	
Chemotherapy + targeted therapy	0 (0)	2 (2.6)	
Chemotherapy + immunotherapy	0 (0)	1 (1.3)	
LCT to metastatic sites			
Surgery	4 (40)	65 (84.4)	.005
Radiation	8 (80)	55 (71.5)	.730
pT			.827
1	2 (20)	19 (24.7)	
2	1 (10)	17 (22.1)	
3	4 (40)	17 (22.1)	
4	3 (30)	24 (31.1)	
pN			.918
0	5 (50)	11 (14.3)	
1	3 (30)	36 (46.7)	
2	2 (20)	30 (39)	
3	0	0	
Differentiation			.498
Well	0	3 (3.9)	
Moderate	4 (40)	24 (31.1)	
Poor	4 (40)	43 (55.9)	
Undifferentiated	2 (20)	7 (9.1)	
Lymphovascular invasion	3 (30)	17 (22.1)	.415
Resection margin			.444
R0	8 (80)	70 (91)	
R1	2 (20)	5 (6.5)	
R2	0	2 (2.5)	
Primary tumor site recurrence	0	11 (14.3)	.239
Local recurrence	1 (10)	22 (28.5)	.272
Distant recurrence	6 (60)	41 (53.2)	.477
30-day survival	10 (100)	77 (100)	1
90-day survival	8 (80)	72 (93.5)	.183
Median PFS, mo	17.7	18.4	.279
Median OS, mo	39.2	37.5	.452

Categorical variables are presented as number (percentage).

LCT, local consolidative therapy; OS, overall survival; PFS, progression-free survival; pN, pathologic nodal status; pT, pathologic tumor status.

Pathologic tumor status (pT) between groups was comparable, although fewer BM individuals had tumors less than pT3 (30% [n = 3/10]). The most common pathologic nodal status (pN) was pN0 (50% [n = 5/10]) in BM patients and pN1 (46.7% [n = 36/77]) in non-BM patients. Moderate and poor tumor differentiation was equally prevalent in BM patients (40% [n = 4/10]), whereas most non-BM patients had poorly differentiated tumors (55.9% [n = 43/77]). Adjuvant therapy was administered to 7 (70%) BM patients and 42 (54.5%) non-BM patients.

### Survival Outcomes

After pulmonary resection, 0 BM patients and 11 (14.3%) non-BM patients experienced primary tumor site recurrence (Table 2). Regional recurrence developed in 1 (10%) BM patient and 22 (28.5%) non-BM patients. Distant recurrence was common in both groups (BM patients, 60% [n = 6/10]; non-BM patients, 53.2% [n = 41/77]). At median follow-up of 18.3 months, median PFS was 17.7 months (95% CI, 5.13-30.29) for BM patients and 18.4 months (95% CI, 11.86-24.86) for non-BM patients (*P* = .28; Figure). All 87 patients survived 30 days after pulmonary resection. The median OS was 39.2 months (95% CI, 11.81-66.51) for BM patients and 37.5 months (95% CI, 22.92-51.98) for non-BM patients (*P* = .45; Figure).

**Figure:**
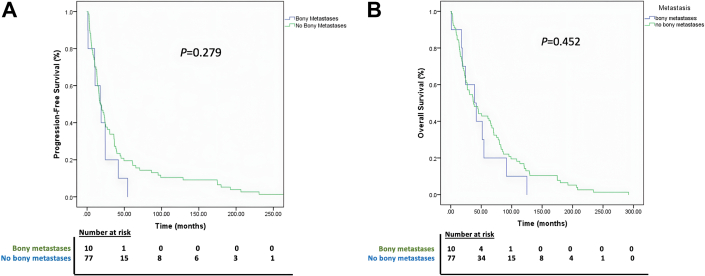
Kaplan-Meier curves comparing (A) progression-free survival and (B) overall survival of groups with and without bone metastases.

Multivariable Cox regression analysis for PFS and OS (Table 3) revealed that BM presence did not independently predict PFS (hazard ratio, 1.45; 95% CI, 0.74-2.82; *P* = .28) or OS (hazard ratio, 1.29; 95% CI, 0.66-2.51; *P* = .45).

**Table 3:** Multivariable Analysis for PFS and OS

Variable	PFS			OS		
	HR	95% CI	*P*	HR	95% CI	*P*
Age at surgery, y	0.994	0.957-1.031	.737	1.011	0.982-1.040	.465
Smoking history	0.409	0.190-1.277	.220	0.748	0.402-1.393	.361
Male sex	0.770	0.356-1.666	.507	0.978	0.554-1.727	.938
pT3-4	1.884	0.912-3.894	.087	1.032	0.605-1.760	.908
pN2-3	0.803	0.366-1.759	.583	0.610	0.326-1.143	.123
2 or 3 metastatic sites	0.447	0.044-4.560	.497	0.700	0.119-4.102	.693
Neoadjuvant therapy	1.572	0.772-3.200	.212	1.304	0.749-2.269	.348
Adjuvant therapy	0.933	0.492-1.771	.833	0.955	0.587-1.554	.853
LCT with surgery	1.780	0.513-6.181	.364	0.599	0.256-1.405	.239
LCT with radiotherapy	2.347	0.900-6.122	.081	1.146	0.552-2.379	.714
Metastatic site						
Bone	1.445	0.740-2.823	.282	1.290	0.662-2.514	.454
Brain	0.752	0.207-2.734	.665	0.622	0.253-1.527	.300
Adrenal	1.185	0.206-6.828	.849	1.040	0.324-3.334	.948
Contralateral lung	1.472	0.360-6.015	.591	0.717	0.251-2.051	.536
Pleura	0.520	0.113-2.386	.400	0.940	0.293-1.143	.123
Lymphovascular invasion	1.708	0.799-3.652	.167	1.152	0.635-2.089	.641

HR, hazard ratio; LCT, local consolidative therapy; OS, overall survival; PFS, progression-free survival; pN, pathologic nodal status; pT, pathologic tumor status.

## Comment

This study evaluated the impact of BM on outcomes in patients with oligometastatic NSCLC undergoing pulmonary resection as part of cLCT. We found that BM and non-BM patients experienced similar OS and PFS outcomes. supporting previous findings regarding the therapeutic benefits of cLCT in these patients.^1^^,^^2^^,^^6^^,^^7^ Notably, BM patients had a higher incidence of R1 resection, although the reason is unclear. Despite our speculation that BM patients harbor more extensive microscopic disease grossly undetectable during surgery, further exploration is warranted for definitive explanations. Nevertheless, our findings support previous investigations regarding the therapeutic benefits of cLCT in patients with oligometastatic NSCLC.^1^^,^^2^^,^^6^^,^^7^

The landmark study by Gomez and colleagues^3^ revealed that LCT prolongs PFS and OS in oligometastatic NSCLC. Subsequent investigations have established lung resection as a valuable LCT modality.^3^^,^^6^ Moreover, cLCT has also been associated with improved OS compared with LCT to only some metastatic sites, which we also demonstrated in our prior work.^2^ These investigations underscore paradigm shifts in stage IV NSCLC treatment, offering opportunities to enhance outcomes.^1^, ^2^, ^3^

As oligometastatic NSCLC management evolves, identifying optimal candidates for surgical consolidation remains imperative.^7^ Our prior work showed diminished cLCT benefits in patients with squamous histologic type, higher intrathoracic stage, and BM.^2^ Historically, BM often precluded LCT, as prior investigations spanned an era during which patients received cytotoxic chemotherapy, with minimal use of targeted therapy. In contrast to our previous findings, we found that PFS and OS were similar regardless of BM presence in this cohort treated with enhanced systemic agents who underwent pulmonary resection. Thus, our current data demonstrate that it may not necessarily be a contraindication to cLCT with or without pulmonary resection. Recent Society of Thoracic Surgeons guidelines emphasize that surgical decisions for stage IV NSCLC, regardless of BM, should involve multidisciplinary discussions, with consideration of patient- and tumor-specific variables.^8^

Patients from prior cohorts tended to be treated with cytotoxic chemotherapy, which has time limits to both tolerability and efficacy. The more contemporary stage IV NSCLC population tends to include oncogene-driven cases treatable with more effective and more tolerable oral agents and patients who are younger with greater performance status. Ultimately, the dogma of the past cannot be applied in the modern setting without carefully assessing the nuances of changing populations of patients, pharmacotherapeutic advances, and surgical innovation.

This study’s limitations include its relatively small sample size of BM patients and the inherent selection bias of those who underwent resection, with the understanding that they were likely to be healthier than those who did not. In addition, the specific number of bone metastatic lesions in BM patients was not captured in our analyses.

To summarize, in this study evaluating patients with oligometastatic NSCLC who underwent pulmonary resection as part of cLCT, we found that BM had no impact on OS or PFS. Our findings corroborate previous reports regarding the benefits of cLCT in appropriately selected patients. As benefits also extend to patients with oligometastatic NSCLC with BM, our data suggest that this population may still be considered for cLCT and lung resection.
